# Effect of microgravity on the feasibility and accuracy of dental procedures

**DOI:** 10.1038/s41526-025-00552-2

**Published:** 2025-12-13

**Authors:** Tine Šefic, Hana Prtenjak, Simon Oman, Aleš Fidler

**Affiliations:** 1 Department of Dental Medicine, Faculty of Medicine, University of Ljubljana, Ljubljana, Slovenia; 2 Faculty of Mechanical Engineering, University of Ljubljana, Ljubljana, Slovenia; 3 Laboratory for Machine Elements, Faculty of Mechanical Engineering, University of Ljubljana, Ljubljana, Slovenia; 4 Department of Endodontics and Restorative Dentistry, Faculty of Medicine, University of Ljubljana, Ljubljana, Slovenia; 5 Department of Endodontics, University Clinical Centre Ljubljana, Ljubljana, Slovenia

**Keywords:** Dental caries, Dentistry

## Abstract

Developing effective countermeasures against oral health risks is essential for long-duration space missions. This study evaluated the feasibility of performing restorative dentistry procedures in a microgravity environment. A parabolic flight campaign aboard the Airbus A310 was conducted through the ESA Academy Experiments programme. The campaign included 90 parabolas over 3 days, each providing ~22 s of microgravity. Two senior dentistry students performed 72 caries preparations and 36 composite restorations on artificial teeth in three environments: ground, microgravity, and steady flight. Accuracy was evaluated using computer-aided 2D image analysis for preparation errors and 3D scanning for restoration errors. Statistical analysis using two-way ANOVA revealed no significant impact of environmental conditions on preparation (*p* = 0.623) or restoration (*p* = 0.139) accuracy, although operator differences were observed. These findings indicate that microgravity does not significantly impair the accuracy of restorative dentistry procedures, highlighting the potential to expand dental treatment in space.

## Introduction

The possible impact of space exploration on oral health was first recognised in the early days of space travel^1^. Since then, the relevance of space dentistry development^2–4^ as an integral part of space medicine^5–8^ has been widely accepted. Dental emergencies in space pose a significant risk to mission success by potentially incapacitating astronauts. Several occasions of spaceflight dental issues have been reported, including crown displacement, dental pain, and dental caries^9^. For a 3-man, 28-day mission, a 0.92% risk was calculated for an in-flight dental event capable of significantly impairing a crew member’s productivity^10^. Furthermore, a dental abscess has been identified as the medical condition most likely to necessitate evacuation from the International Space Station^11^. Although the stringent selection criteria may reduce the probability of medical events during short-duration missions, this advantage diminishes after 42–180 days^12^. As space exploration progresses, mission durations also increase, with anticipated missions to Mars expected to last between 500 and 700 days. Lengthy missions in combination with changes in the oral cavity^3^ and decreased motivation^7,13^ may lead to the development of new dental pathologies or worsening of the existing ones. Additionally, research indicates that microgravity alters *Streptococcus mutans* gene expression, potentially altering its cariogenic potential during spaceflight^14,15^. Such a situation calls for preparedness to perform dental procedures in a microgravity environment in space^2^.

Microgravity is the main factor affecting the performance of medical procedures in space. It affects psychomotor tasks^16–19^ and requires adaptation of the surgical workstation to the specific requirements^20,21^. So far, the feasibility of medical procedures has been evaluated in a microgravity environment with non-conclusive results, reporting comparable^22,23^ or inferior^24–26^ performance of surgical procedures. The adaptation of the surgical workstation was reported, aiming to comply with the safety requirements^20,21^ and space limitations^27^. Recently, the use of robots was also considered^28^, but communication quality and delays ranging from 4 to 24 min pose significant challenges. Despite the anticipated need for dental procedures in space, there is no available data on dental workstation design or the accuracy of dental procedures in microgravity.

The aim of the study was two-fold: (a) to construct the simulated dental workstation (SDW) and (b) to evaluate the accuracy of simulated dental procedures in microgravity (MG), steady flight (SF), and ground (GND) environments.

## Methods

The study received approval from the National Medical Ethics Committee of the Republic of Slovenia (Numbers: 0120-206/2023/6 and 0120-206/2023/9). The 83rd ESA Parabolic Flight campaign occurred from November 20 to December 1, 2023, at Bordeaux-Mérignac Airport.

### Operators and Simulated Dental Workstation

Three senior dentistry students with 3 years of patient care experience volunteered for the experiment and signed an informed consent form. They had to complete the parabolic flight medical examination, have a history of high tolerance for motion-induced discomfort, be right-handed, and had to agree to take 0.175 mg of scopolamine, which was administered half an hour before flight to prevent motion sickness.

**Fig. 1:**
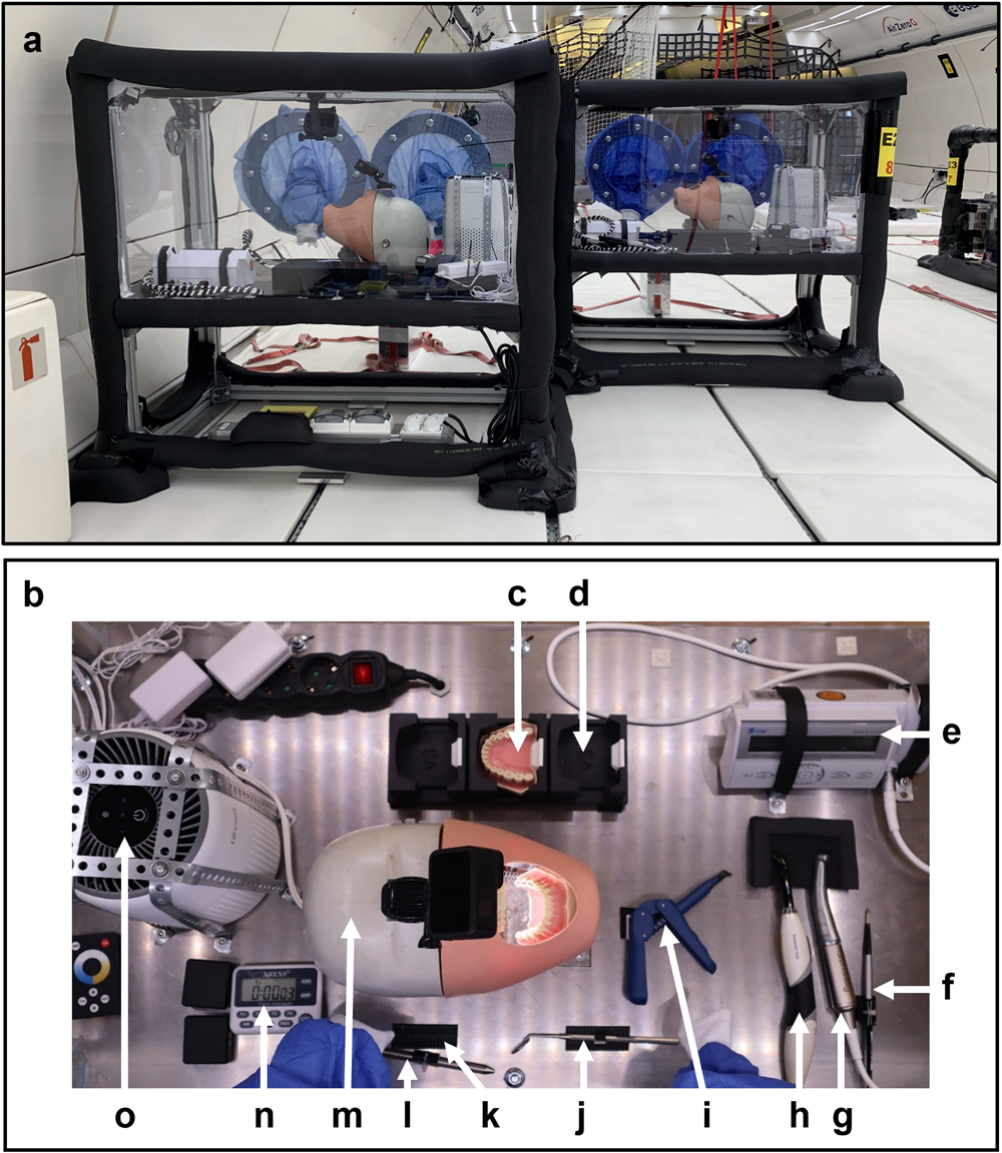
Simulated Dental Workstations (SDW) with dental operatory setup. **a** Front view of the SDWs on board the Air Zero G plane. **b** Bird’s-eye view of dental instruments, materials, and devices. **c** Mandibular teeth (Frasaco, Tettnang, Germany). **d** 3D-printed protection case. **e** Battery-powered endodontic motor (VDW.GOLD Reciproc; VDW, Munich, Germany). **f** Painting brush with fine bristles for eliminating plastic debris from the cavity during preparation (Golden Stag, Dynasty, New York, USA). **g** Contra angle handpiece (Synea WA-56LT, W&H Dentalwerk, Bürmoos, Austria). **h** Polymerisation light (Bluephase G4, Ivoclar Vivadent, Schaan, Liechtenstein). **i** Applicator with dental composite (X-Tra fill bulk, Voco, Cuxhaven, Germany). **j** Dental mirror (Cone socket mirror single sided, Hu-Friedy, Chicago, USA). **k** Fastener for instruments (Dual Lock, 3M, Maplewood, Minnesota, USA). **l** Composite instrument (W3, Hu-Friedy, Chicago, USA). **m** Dental manikin (Frasaco, Tettnang, Germany). **n** Timer for steady flight intervals (XREXS, Delaware, USA). **o** Air purification system (Levoit Core Mini LAP-C161, LEVOIT, California, USA). (Photo: SpaceDent).

To standardise the workstation setup, students of similar height were selected. Extremity measurements were taken, and a full-scale (1:1) mock-up was built with dimensions adjusted to the group’s mean height and extremity measurements. After validating the mock-up, two identical Simulated Dental Workstations (SDWs) (Fig. 1a) were designed and produced in accordance with the technical and safety requirements for parabolic flights (Novespace, Bordeaux, France). Safety inspection and approval of the SDWs were performed 41, 8, and 4 days before the flight, with final inspection occurring after their installation on board the dedicated aircraft (Airbus A310 Zero G, Novespace). Closed structures with transparent walls, each featuring two perforations fitted with plastic sleeves for access, contained a dental manikin (Fig. 1m), a battery-powered dental drill (Fig. 1e), dental instruments and dental materials for simulated procedures (Fig. 1b). The structure also included a video recording system, an air filtration system (Fig. 1o) and a LED illumination system.

### Training of operators

The three operators underwent structured training and performed 8 training sessions on the workstation prior to the attachment on the plane, as well as 8 training sessions on the plane prior to the flight. During the training sessions, which lasted 2.5 h (same as the duration of the flight), operators performed the procedures, guided by a video simulation of the flight. The video simulation also incorporated parabolic-flight audio, including pilot announcements of parabola stages over the cabin speaker, and parabola count. All trainings were performed with teeth, instruments, and materials identical to those used in the experiment.

### Aircraft and flight

**Fig. 2:**
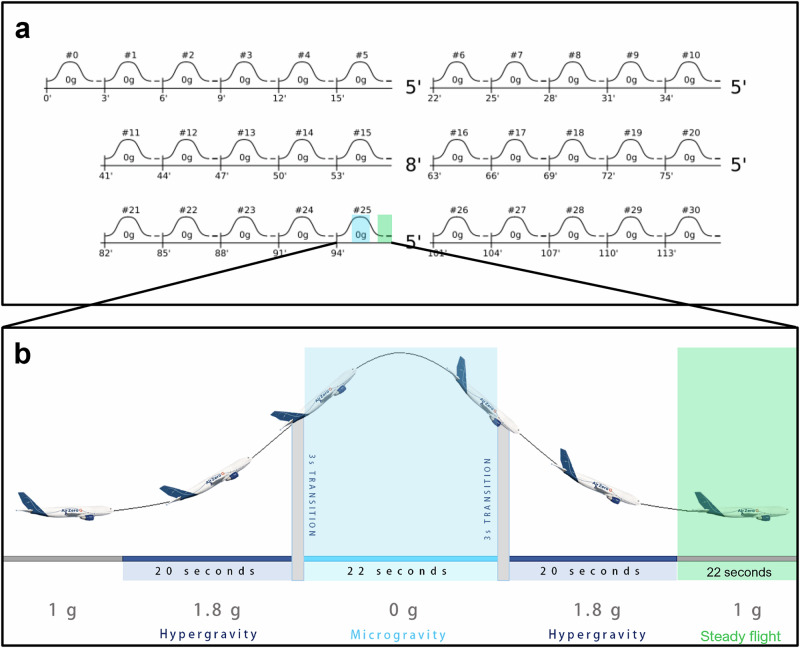
Description of the parabolic flight. **a** Each flight included 30 parabolic manoeuvres, grouped in sets of five parabolas, with each set followed by a 5- or 8-min interval of steady flight. **b** Each parabola consisted of two 20-s intervals of hypergravity with a 22-s interval of microgravity (indicated by the light blue area) in between. Between parabolas, there was a 100-s interval of steady flight. Dental procedures were conducted during the microgravity phase and timed 22-s intervals during the steady flight (indicated by the green area) (Photo: Reproduced with permission from ©Novespace and ©ESA, with modifications).

In each of three consecutive days, a 2.5-h parabolic flight was performed. Each flight provided 30 parabolas (Fig. 2a) with 22-s microgravity (MG) intervals and an equal number of 22-s steady flight (SF) intervals between the parabolas (Fig. 2b). During the flight, two operators conducted the experiment while a third served as backup in case of motion sickness. The two operators performed caries preparations (Days 1 and 2) and composite restoration placement (Day 3) procedures on the dental manikin’s artificial teeth. The ground (GND) environment procedures were performed while the aircraft was stationary on the runway. To match the MG and SF timing conditions, the procedures were performed during 90 intervals, each lasting 22 s.

In the SF and MG intervals during the flights, the number of on-board experiments (*n* = 11), experimenters (*n* = 40), temperature (17–20 °C) and humidity (<15%) were constant, while the vibration level, noise level (MG: 70–80 dB) and residual gravity (MG: *Z*-axis = 0 ± 0.03 g, *X*-, *Y*-axes = 0 ± 0.02 g; SF: *Z*-axis = 1 ± 0.05 g, *X*-axis = 0 ± 0.04 g, *Y*-axis = 0 ± 0.02 g) were dependent on the flight phases. During the GND intervals, the number of people (*n* = <15), temperature (10–20 °C), humidity (70–100%) and noise (produced by people) were situational, while vibrations and accelerations were absent.

### Dental procedures

**Fig. 3:**
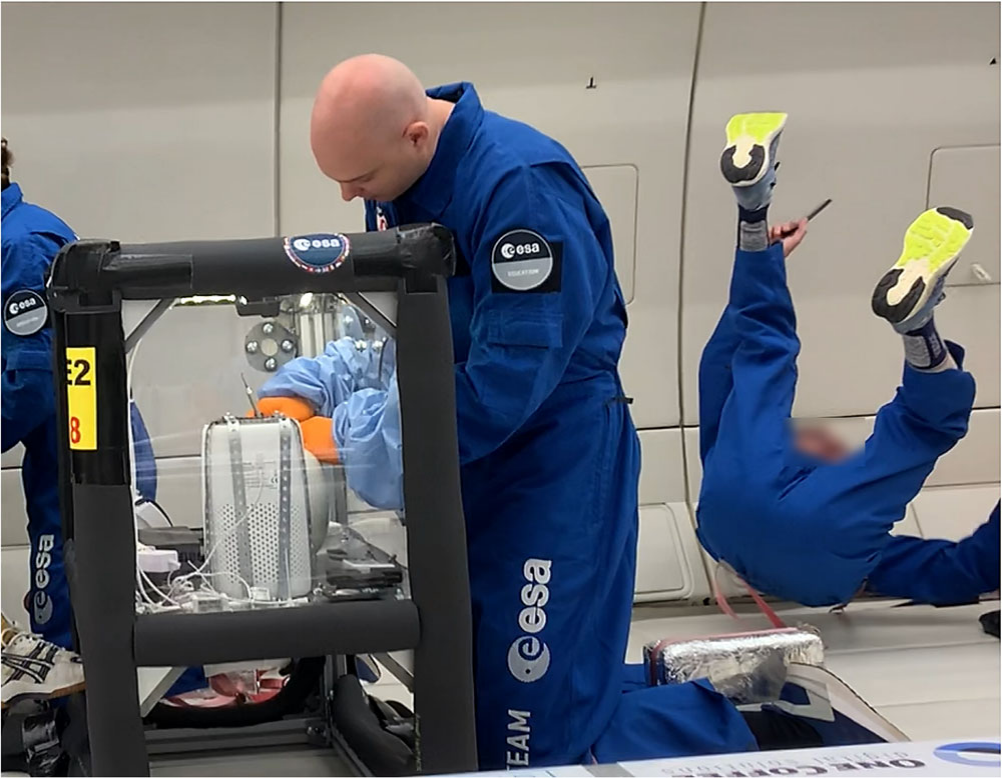
Depiction of an operator’s position during caries preparation and composite placement procedures. The practitioner is shown in a 9 o’clock kneeling position. To maintain this position during the parabolic flight manoeuvre, the practitioner’s legs were strapped at the knees. On the right side of the image, another researcher is experiencing microgravity. (Photo: Reproduced with permission from ©ESA, with modifications).

**Fig. 4:**
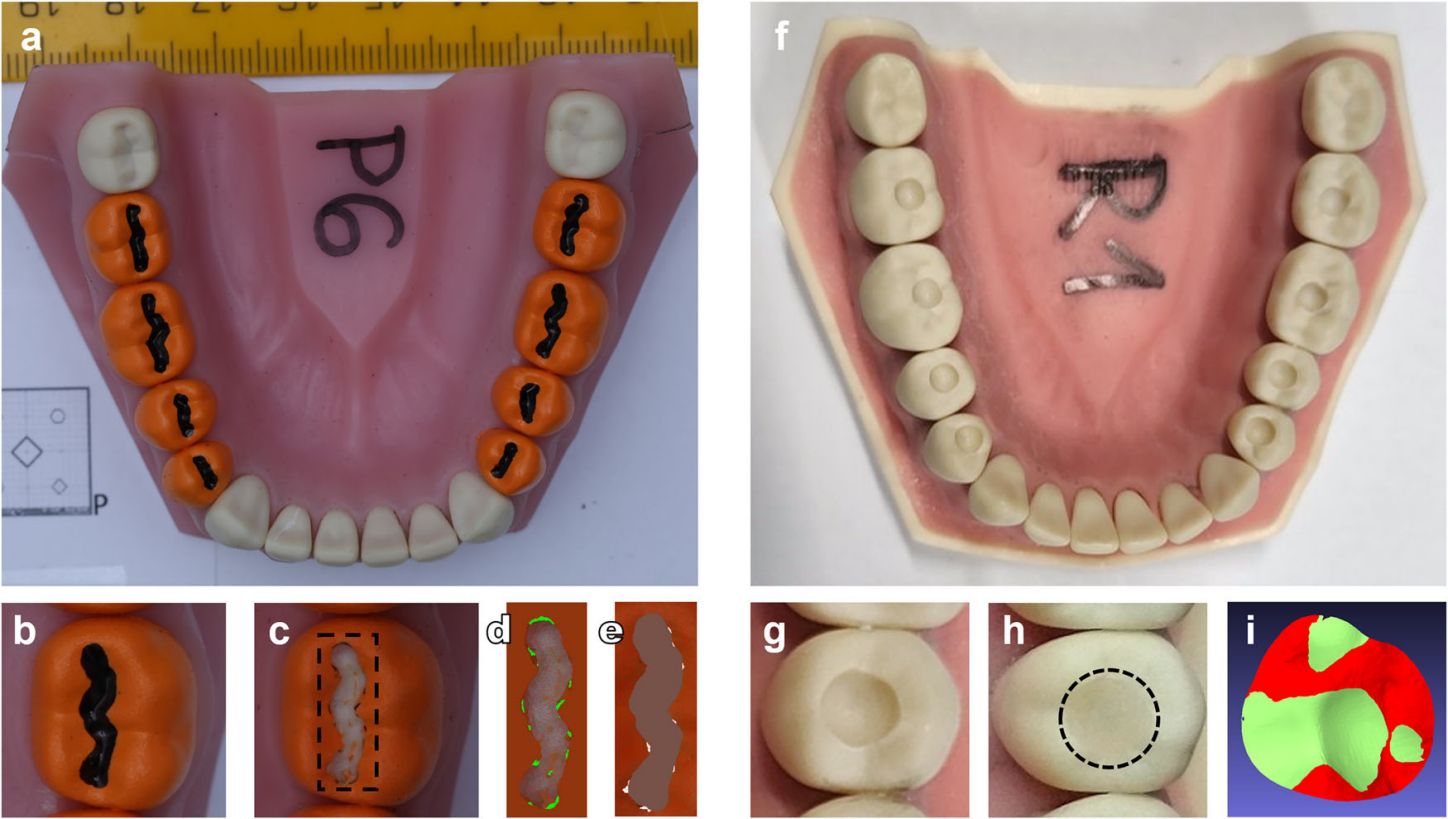
This image series illustrates the sequence and analysis of caries preparation and composite restoration procedures. **a** Coloured teeth for the preparation procedure with simulated caries lesion (indicated by a black delineation) and intact enamel (marked by orange colouring). **b** A visual representation of a molar (tooth 36) before and **c** after preparation procedure. A dashed square indicates the area featured in images (**d**) and (**e**). **d** A visual representation of a computer-aided evaluation of under-preparation (indicated by green) and **e** over-preparation (indicated by white). **f** Plastic mandibular teeth for the restoration procedure with standardised cavities. **g** A visual representation of a premolar (tooth 35) with a standardised cavity before and **h** after restoration procedure. A dashed circle indicates the area shown in image (**i**). **i** Additionally, a visual representation of an under-fill (indicated by light green) and over-fill (indicated by red) composite placement relative to the baseline morphology of the tooth. (Photo: SpaceDent).

Dental procedures were performed using direct vision in a straightened kneeling 9 o’clock position during all phases of the experiment. (Fig. 3; A written informed consent was obtained for the publication.) Operators’ hands were inserted through two openings with plastic sleeves. For each operator, 12 simulated caries preparations and 6 composite restoration placements were planned in each of the three environments. The procedures were performed on equally distributed mandibular molar and premolar (Fig. 4a, f) plastic teeth (Frasaco, Tettnang, Germany). The teeth were prepared before the flight according to the simulated dental procedure.

### Simulated caries preparation

To standardise cavity preparations, we used plastic teeth with identical, well-defined occlusal morphology. Firstly, the crown surfaces of teeth (*n* = 72) were lightly sandblasted and coloured with orange matte spray paint to simulate intact enamel (Fig. 4a). Carious lesions were simulated by delineating occlusal fissures with a 1.5 mm wide water-resistant black marker (No. 317-9, Staedtler, Nuremberg, Germany) (Fig. 4b). Because the fissure was narrower than the standardised dental bur, gaining access required the removal of approximately 3 mm of the adjacent cuspal structure. Therefore, preparation depth was determined by cusp–fissure anatomy. A DSLR camera (Canon EOS 40D, Tokyo, Japan) with a macro lens (Canon EF-S 60mm, Tokyo, Japan) was used to acquire baseline occlusal images. The camera was mounted on a rigid stand to ensure a constant position, distance, and angle in relation to the mandibular jaw with teeth. A uniform and replicable illumination of the samples was provided by an indirect illumination from a flash unit, which was directed away from the object of interest and thus evenly illuminated the entire laboratory room in which the photographs were taken. Otherwise, the laboratory room was darkened during photography.

During the first two flights, each operator was tasked with performing occlusal preparations in a standardised sequence. The preparation on each tooth had to be completed during five 22-s intervals. Using a contra-angle with a round 1.2 mm diameter carbide bur (Fig. 1g), a surface of simulated caries lesion (black) was intended for removal while avoiding simulated intact enamel (orange). The painting brush (Fig. 1f) or dental mirror (Fig. 1j) was alternately held in the left hand for either removing plastic debris from the cavity or inspecting the residual carious lesion. To enable the immediate commencement of the preparation upon transition from hypergravity to the microgravity phase, the dental handpiece was held during the hypergravity phase. To match the MG and SF timing conditions during the GND phase, the preparations were performed in a standardised sequence during 60 intervals, 22 s in length.

After the campaign, the occlusal images were acquired again, and the photographs were registered to the baseline images using ImageJ Fiji software (version: 1.54f https://imagej.net/software/fiji/)^29^ utilising “Register Virtual Stack” tool^30^. The registered images were overlaid in the Krita programme (version: 5.2.2, https://krita.org/en/). Using the crop method (Fig. 4c), composite baseline and follow-up images of each tooth’s occlusal surfaces were isolated and exported to the REBMIX programme (version: 2.15.0, (https://cran.r-project.org/web/packages/rebmix/index.html). The segmentation of the imported baseline and follow-up images was performed using the REBMIX programme (Version 2.15.0, https://cran.r-project.org/web/packages/rebmix/index.html)^31,32^. Prior to segmentation, the images were processed to enhance the resolution of the transitions between the individual colour tones. Subsequently, the Gaussian multimodal distribution of the colour tones at the pixel level was determined using the REBMIX programme. Based on the mixture distribution obtained, clusters were formed for each distribution component. The number of components of the mixture distribution was manually selected based on the number of visible colours. In this particular case, three colours were tracked: the original tooth colour (not in all images), black, and orange. Each colour was then initialised at the centre of the associated distribution component. If more than one region of the same colour was present, further clusters were formed. The result was that the number of pixels in each cluster was exported together with the cluster properties (i.e., which colour it belongs to, the exact position of the pixels in the cluster).

For preparation evaluation, clusters were formed by combining corresponding parts of the images. In the baseline image, two clusters were formed, namely “caries”—the caries area, represented by black colour—and “intact”—the intact area, represented by orange colour. In the follow-up images, two further clusters were formed, namely “underpreparation”—the unprepared area, represented by the remaining black colour within the caries area (Fig. 4d), and “overpreparation”—the prepared area within the intact area, represented by native tooth colour (Fig. 4e). The pixels for the “overpreparation” region were calculated by first summing the “black” clusters and the “tooth-coloured” clusters of the “after preparation” image (Fig. 4c) and subtracting the “black” cluster area of the baseline image (Fig. 4b). Based on the number of pixels in each cluster obtained, preparation error was calculated by the equation (Eq. (1)):

1 $${\rm{Preparation}}\;{\rm{error}}=\frac{{\rm{underpreparation}}({\rm{no}}.{\rm{of}}\;{\rm{pixels}})+{\rm{overpreparation}}({\rm{no}}.{\rm{of}}\;{\rm{pixels}})}{{\rm{caries}}({\rm{no}}.{\rm{of}}\;{\rm{pixels}})}$$

### Composite restoration placement

Three sets of teeth with standardised occlusal cavities (Fig. 4f) were prepared for each operator. At first, the crown surfaces were lightly sandblasted to enhance scanning accuracy. Baseline teeth morphology was captured using a wireless intra-oral scanner (TRIOS 4, 3Shape, Copenhagen, Denmark). Thereafter, a 3.1 mm diameter round carbide dental bur and a Universal Milling Machine (URS-1) with an *x*–*y* table were utilised to standardise the size, location, and depth of the cavities between the sets (Fig. 4g). After cavity preparation, the teeth were scanned again. The average molar cavity volume was 9.75 mm³, while the average premolar cavity volume was 13.10 mm³.

During the third flight, each operator was tasked with performing occlusal composite restorations (Fig. 4h) in a standardised sequence using an oblique layering technique^33^. Restorations on each tooth had to be completed during five 22-s intervals. Using an applicator (Fig. 1i) and composite adaptation instrument (Fig. 1l), the composite layer was applied on the buccal side of the cavity during the first two parabolas and on the lingual side and occlusal surface during the final three parabolas. A 20-s light polymerisation was performed after the second and fifth parabola during steady flight. After the campaign, the teeth were scanned again to acquire the shape of the composite restorations. To match the MG and SF timing conditions during the GND phase, the restorations were performed in a standardised sequence during 30 intervals, 22 s in length. The restoration scans were performed after the campaign.

Scans in STL format were imported into Meshmixer (Version: 3.5.474, Autodesk Inc., San Francisco, CA, USA) for further analysis. Local best fit was achieved using the “Transform” function and a precise fit by separately aligning the cavity and restoration scans to the baseline for each tooth using the “Brush Mode” and “Align to Target” function. Aligned scans of each tooth were isolated using the “Edit: Plane Cut function”, saved in the STL Binary Format (.stl) and imported to the MeshLab (Version 2023.12, https://www.meshlab.net/). Subsequently, the restoration area of each individual mesh was centred with a cylinder (diameter: 3.5 mm) and intersected using the “Mesh Boolean: Intersection” function. Thereafter, the baseline, cavity and restoration mesh were subtracted to acquire the cavity volume (Eq. (2)), underfill (Eq. (3)), and overfill (Eq. (4)) mesh.

2 $${\rm{Cavity}}\;{\rm{Volume}}\;{\rm{Mesh}}={\rm{Baseline}}\;{\rm{Mesh}}-{\rm{Cavity}}\;{\rm{Mesh}}$$

3 $$Underfill\,Mesh=Baseline\,Mesh-Restoration\,Mesh$$

4 $$Overfill\,Mesh=Restoration\,Mesh-Baseline\,Mesh$$

To ensure the integrity of the mesh, the “Remove Isolated Pieces (wrt. Face Num.)”, “Repair Non-Manifold Edges” and “Close Holes” functions were applied. Finally, the volumes of the cavity and restoration underfill and overfill were measured using the “Compute Geometric Measures” function (Fig. 4i). The restoration error was calculated by the equation (Eq. (5)):

5 $$Restoration\,error=Underfill\,volume+Overfill\,volume$$

#### Statistical analysis

A two-way ANOVA was conducted to examine the effects of the environment and operator on preparation and restoration accuracy. Data are mean, 95% CI, unless otherwise stated. Residual analysis was performed to test the assumptions of the two-way ANOVA. Outliers were assessed via inspection of a boxplot; normality was assessed using Shapiro–Wilk’s normality test, and homogeneity of variances was assessed by Levene’s test. Statistical software (IBM SPSS 29.0.0.0) was used for statistical analysis.

## Results

During the three parabolic flights, both operators reported no sickness or any other adverse effects; therefore, there was no need to include the backup operator. There were no technical problems with the experimental setup, and all the procedures were completed as planned within the time limitations. In total, 72 preparations and 36 restorations were completed.

### Simulated caries removal

**Fig. 5:**
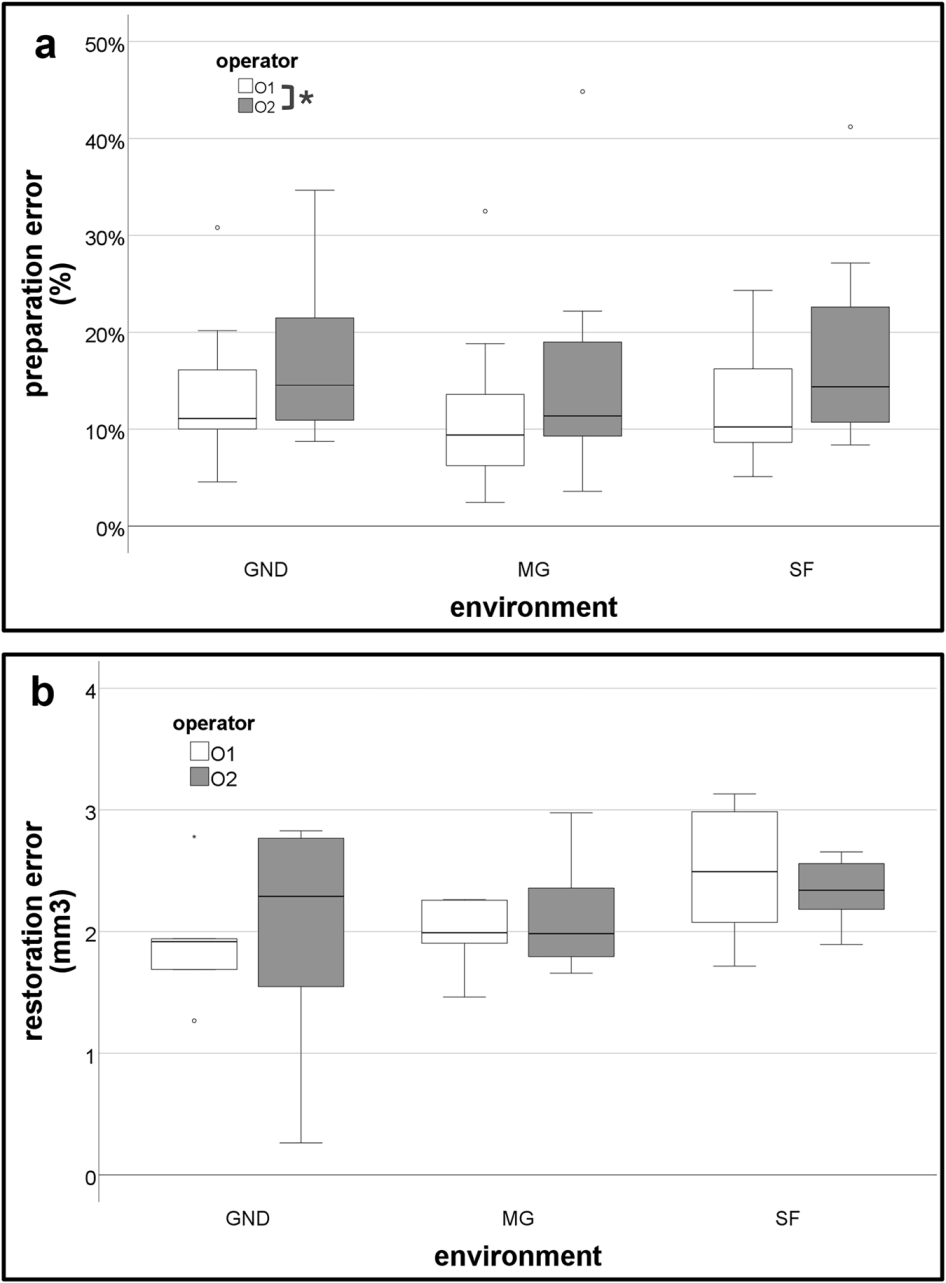
Effect of environmental conditions and operator on the accuracy of dental procedures. **a** Box–Whisker plot for preparation error, evaluated with % of deviation from lesion (mm^2^). GND ground, MG microgravity, SF steady flight. o, *a statistically significant main effect of operator (*p* = 0.036). **b** Box–Whisker plot for restoration error, evaluated with volume difference (mm^3^). GND ground, MG microgravity, SF steady flight. (Photo: SpaceDent).

In the analysis of preparation errors, there were four outliers assessed as being greater than 1.5 box-lengths from the edge of the box in a boxplot (Fig. 5a). Preparation-error measurements are provided in Supplemental Table 1. Residuals were normally distributed (*p* > 0.05) for three groups (O1 GND, O1 SF, and O2 GND), and there was homogeneity of variances (*p* = 0.714). There was no statistically significant interaction effect between environment and operator (*p* = 0.072). A statistically significant main effect of operator (*p* = 0.036) was found, but no statistically significant main effect of environment (*p* = 0.623). No post-hoc test was required.

### Composite restoration placement

In the analysis of restoration errors, there were 2 outliers in the O1 GND group assessed as being greater than 1.5 box-lengths from the edge of the box in a boxplot (Fig. 5b). Residuals were normally distributed (*p* > 0.05) for all groups, and there was homogeneity of variances (*p* = 0.066). There was no statistically significant interaction effect between environment and operator (*p* = 0.791), main effect of operator (*p* = 0.897) and environment (*p* = 0.139). No post-hoc test was required.

## Discussion

The experiment demonstrated that the proposed simulated dental workstation enabled a successful completion of planned dental procedures, with procedural accuracy remaining unaffected by gravitational conditions. This represents an important step towards ensuring effective dental care during long-duration space missions and extra-terrestrial habitation.

The simulated dental workstation, approved by Novespace before flight, allowed sufficiently accurate dental procedures, including simulated caries preparation and composite restoration. There was no significant difference in performance between MG, SF, or GND environments. Both operators successfully completed all the planned procedures, consisting of 72 preparations and 36 restorations, within the time limits in each environment. A significant difference in preparation errors was observed between the two operators, with error rates of 12.58% and 16.79%, while there was no significant difference between operators in restoration errors. As the interaction between operators and the environment was not statistically significant, differences between operators might reflect individual operator variability.

It has been demonstrated that several medical procedures, including microsurgery^23^, can be performed in microgravity conditions. However, compared with ground conditions, parabolic flight was associated with higher forces applied to the instruments and lower knot quality^26^. In laparoscopic procedures, efficiency was decreased, and injury to the simulated tissues was increased^22^. This is in contrast with our results, showing no reduction in accuracy for both preparation and restoration procedures. This discrepancy may be attributed to the fact that dentists stabilise their hand movements by positioning their fingers to the surrounding structures, predominantly teeth, but also the cheek or chin during dental procedures. Additionally, the fixed position of the dental manikin head might provide further support and stabilisation. Therefore, with a real dental patient, stabilisation of the patient’s head and torso should be implemented. Using additional stabilisation structures, such as a rotating arm and knee rest, as well as supplementary hand supports, could enhance the accuracy and safety of other surgical procedures. In addition, to accommodate left-handed operators, our workstation’s central plate can be rotated 180°. Furthermore, to save space, an inflatable microgravity surgical workstation design was proposed independently from our study, but has not been flight-tested so far^21^.

Experimental opportunities on the Air Zero G plane are limited; thus, the number of operators performing the experiment during the parabolic flight was limited to two. However, the number of preparation and restoration procedures per operator was high in comparison to other studies due to careful planning, simplification and optimisation of procedural steps, which would also be relevant for real situations. To reduce the differences between in-flight and on-the-ground cabin conditions, on-the-ground control procedures were performed half an hour after the flight, immediately following the post-flight briefing. Furthermore, the use of an isolation chamber made fluctuations even less prominent.

The operators’ kneeling posture enabled a comfortable workflow and could be suitable for extended procedures. During the experiment, the operators performed the procedures in the 9 o’clock position, which is often an introductory position for dental students and could be used for astronauts with limited dentistry experience. This position enables direct dental treatment, simplifies instrument handling, and could potentially be adopted as one of the preferred options for dental treatment in space. In contrast, indirect, mirror-mediated work is more challenging and requires better coordination. Furthermore, due to prolonged exposure to the space environment and physiological changes (i.e., altered visuospatial perception, reduced sensorimotor performance, Spaceflight Associated Neuro-ocular Syndrome (SANS) related visual changes, etc.)^34–36^, the difficulty of performing dental procedures might increase with time. Therefore, simplifying the workflow might be of utmost importance. Given the low expected frequency of dental problems in space, any ergonomic trade-offs of direct work are likely minor.

Due to the parabolic-flight provider’s safety requirements, the use of water was avoided, as it would require a double-layered workstation, along with an additional high-capacity suction system and a watertight electrical system for our experiment. These measures would complicate the experiment and introduce additional variables, limiting our ability to foresee all potential risks. Due to these restrictions, a slow-speed dental bur was preferred. This choice had two additional benefits. First, the slow-speed bur cut the plastic material rather than grinding it, creating a clearer distinction between the tooth colouring and the preparation surface, which improved the image analysis. Second, a slow-speed dental bur could be used with a transportable, battery-powered Reciproc unit paired with a 1:1 contra-angle operating at 10,000 rpm. The Reciproc unit met the parabolic-flight safety standards and simplified our preparation setup.

Another concern is the selection of restorative material. The use of conventional or resin-modified glass ionomers requires mixing before application, which is challenging in microgravity, while conventional composites require application and drying of the adhesive, both complicating the dental procedure. During parabolic flight, before polymerisation, operators performed composite sculpting through several microgravity phases with intermittent hyper-gravity phases. During hyper-gravity phases, the low-viscosity composites might be susceptible to shape modification, rendering the results unrepresentative and skewing the results of the experiment. Consequently, regular viscosity composites were used, although low viscosity dental materials might be used in space.

In microgravity, surface tension causes water to coalesce into globules, allowing small volumes to be handled safely (for example, during routine toothbrushing). Problems arise when water is delivered under pressure, much like a dental water spray, which can disperse droplets throughout the cabin. Therefore, residuals like carious debris and etchant can be removed using methods that do not disperse water droplets, such as pre-wetted sponges, viscous water gels, or targeted syringe irrigation, used in conjunction with continuous, high-capacity suction. On Earth, water spray is typically the primary clearing method, and suction is secondary. In microgravity, for safety reasons, this paradigm may be reversed, with suction as the primary modality and limited, targeted water application as secondary aid. Using this approach, residuals can be removed while minimising free-fluid dispersion.

Similarly, due to the surface tension, saliva is expected to remain accumulated within the oral cavity during longer dental procedures. Given the locations of the parotid, submandibular, and sublingual glands, most saliva may accumulate in the oral vestibule and beneath the tongue. In addition, due to the absence of gravity, it is expected that saliva will not flow toward the oropharynx. Excess saliva can therefore be removed periodically with a suction system from the oral vestibule and beneath the tongue. Gauze pads may also be used, but this would introduce additional waste in the limited spacecraft environment. Another possibility for saliva control is to use a rubber dam. However, small gaps between the dam and the lips may persist. A rubber dam may also serve as a countermeasure against aspirating small dental parts, as this can present an increased risk in microgravity. In addition, isolation of a patient’s head with a neck seal similar to that of a dry suit seal could be utilised; however, caution should be applied to prevent vascular implications resulting in dizziness, lightheadedness, or even syncope^37^.

In contrast to the conventional dental practice environment, where the operator’s body remains stable, and the hands are the primary moving parts, in microgravity, the entire body tends to drift or rotate during procedures. These movements are gradual but noticeable, and the operator must remain mindful of them to make continual adjustments. Therefore, procedures in microgravity should be performed at a slower pace, avoiding sudden or jerky movements, as these can set the whole body in motion. A kneeling position with a strap behind the knees provided enough stability to maintain a comfortable workflow.

Operator 1 observed that movements were initiated from the torso, with the torso serving as the primary anchor, rather than relying on effort against gravity as on Earth. Downward movements had to be consciously controlled, unlike on Earth, where gravity naturally assists.

Operator 2 added that many dentists assume that every arm movement needs to have support or else mistakes will happen, but this is not true. Microgravity is a gentle environment, and it will not force you into movements you do not want to make. You can get into position and start the procedure.

In microgravity, using a foot pedal to activate the dental handpiece is difficult. Therefore, we replaced it with an on-handpiece pressure button. For safety, the drill operated only while the button was held and shut off upon release. This prevented the dental drill from being active in the event of release in microgravity. Operators reported no difficulty performing preparations while pressing the button. However, during longer procedures, maintaining continuous pressure would lead to finger fatigue, operators reported.

Given the successful simulation of the dental procedures and with careful consideration of safety and environmental conditions, further validation of the results on a similar model or with a human patient should be performed in the future.

In the past, human spaceflight operations were largely confined to the International Space Station (ISS), where proximity to Earth made emergency evacuation feasible. Consequently, dental care was limited to emergency measures. As we prepare for long-duration missions to Mars, evacuation to Earth will no longer be possible. Therefore, in-mission dental capabilities must expand beyond first aid to include preventive care, restorations, endodontic treatment, and, as a last resort, extractions.

In conclusion, the conducted study provides valuable insights into the practicability of performing dental and potentially surgical procedures using burs or shaping instruments. With comprehensive pre-mission training, as indicated by dentistry students, dental instruments could be utilised safely and effectively. This opens the potential to broaden the range of medical interventions that could be carried out in space.

## Supplementary information

Supplemental Table

## Data Availability

The datasets generated and analysed during the current study are available from the corresponding author upon reasonable request.
