# Supplementary material for: Effect of microgravity on the feasibility and accuracy of dental procedures
Source: NPJ Microgravity. 2025 Dec 13;12:9. doi: 10.1038/s41526-025-00552-2 (PMC12808107; doi:10.1038/s41526-025-00552-2)
Supplement: Supplementary file 1 — Supplemental Table [file 41526_2025_552_MOESM1_ESM.docx]

| Operator  (1,2) | Environment  (Microgravity, Steady flight, Ground) | Preparation error  (%) |
| --- | --- | --- |
| 1 | Microgravity | 2.44 |
| 1 | Microgravity | 8.98 |
| 1 | Microgravity | 18.83 |
| 1 | Microgravity | 14.42 |
| 1 | Microgravity | 12.78 |
| 1 | Microgravity | 12.72 |
| 1 | Microgravity | 5.56 |
| 1 | Microgravity | 6.93 |
| 1 | Microgravity | 4.24 |
| 1 | Microgravity | 32.49 |
| 1 | Microgravity | 9.82 |
| 1 | Microgravity | 8.24 |
| 1 | Steady flight | 15.38 |
| 1 | Steady flight | 14.77 |
| 1 | Steady flight | 8.11 |
| 1 | Steady flight | 24.33 |
| 1 | Steady flight | 10.03 |
| 1 | Steady flight | 10.41 |
| 1 | Steady flight | 6.01 |
| 1 | Steady flight | 10.04 |
| 1 | Steady flight | 21.26 |
| 1 | Steady flight | 5.10 |
| 1 | Steady flight | 9.19 |
| 1 | Steady flight | 17.09 |
| 1 | Ground | 20.17 |
| 1 | Ground | 11.75 |
| 1 | Ground | 15.58 |
| 1 | Ground | 30.80 |
| 1 | Ground | 16.68 |
| 1 | Ground | 14.31 |
| 1 | Ground | 4.56 |
| 1 | Ground | 9.03 |
| 1 | Ground | 10.28 |
| 1 | Ground | 10.47 |
| 1 | Ground | 9.78 |
| 1 | Ground | 10.33 |
| 2 | Microgravity | 16.52 |
| 2 | Microgravity | 18.60 |
| 2 | Microgravity | 5.91 |
| 2 | Microgravity | 10.00 |
| 2 | Microgravity | 22.20 |
| 2 | Microgravity | 10.23 |
| 2 | Microgravity | 10.14 |
| 2 | Microgravity | 8.59 |
| 2 | Microgravity | 44.84 |
| 2 | Microgravity | 19.40 |
| 2 | Microgravity | 3.59 |
| 2 | Microgravity | 12.51 |
| 2 | Steady flight | 15.59 |
| 2 | Steady flight | 10.97 |
| 2 | Steady flight | 27.16 |
| 2 | Steady flight | 10.46 |
| 2 | Steady flight | 41.20 |
| 2 | Steady flight | 13.06 |
| 2 | Steady flight | 23.70 |
| 2 | Steady flight | 10.08 |
| 2 | Steady flight | 19.53 |
| 2 | Steady flight | 13.16 |
| 2 | Steady flight | 21.51 |
| 2 | Steady flight | 8.37 |
| 2 | Ground | 34.66 |
| 2 | Ground | 13.95 |
| 2 | Ground | 20.47 |
| 2 | Ground | 10.61 |
| 2 | Ground | 15.14 |
| 2 | Ground | 13.14 |
| 2 | Ground | 22.52 |
| 2 | Ground | 8.75 |
| 2 | Ground | 20.27 |
| 2 | Ground | 11.27 |
| 2 | Ground | 26.43 |
| 2 | Ground | 9.93 |

Supplemental Table 1: Preparation error measurements (%) by operator (operator 1 and operator 2) and environment (microgravity, steady flight, ground).
